# Impact of caregiving burden on family caregivers of older adults with chronic diseases

**DOI:** 10.3389/fpsyt.2026.1785065

**Published:** 2026-04-28

**Authors:** Fahad A. Alghamdi

**Affiliations:** Faculty of Nursing, Al-Baha University, Al-Baha, Saudi Arabia

**Keywords:** caregiver burden, chronic diseases, family caregivers, family support, older adults, Saudi Arabia, Zarit Burden Interview

## Abstract

**Objective:**

With the worldwide aging population, chronic diseases are increasing, resulting in growing dependency on informal caregivers. Caregiver burden, a complex stress constituting physical, psychological, social and financial strains, is one of many public health problems. Despite its importance, few studies have been conducted on the prevalence and predictors of caregiver burden in Saudi Arabia. This study aims to address this gap by evaluating the impact of caregiving burden of family members who take care of older relatives with chronic diseases.

**Methods:**

Family caregivers for older adults were enrolled in a cross-sectional study in the Al-Baha region of Saudi Arabia. A structured questionnaire incorporating the Zarit Burden Interview was used to collect data, including demographic and caregiving characteristics. Descriptive statistics such as frequencies, percentages and chi-square, and inferential testing were used to predict caregiver burden using SPSS.

**Results:**

About one-third of caregivers had a moderate-to-high burden, with 36.36% reporting mild to moderate burden and 27.27% high burden. Bivariate analyses indicated that caregiver gender, hours of caregiving, and family support were significantly associated with burden (p < 0.05). Female caregivers and those providing 2–3 hours of care per day reported higher burden. Caregivers with low family support were more likely to experience high burden (51.9%) compared with those with high support (11.2%). However, multivariable ordinal logistic regression revealed that relationship to the care recipient was the only independent predictor of caregiver burden (OR = 22.48, 95% CI = 4.09–123.46, p < 0.001), whereas gender, age, education, marital status, and occupation were no longer significant.

**Conclusion:**

Family caregivers of older adults with chronic diseases in Al-Baha experience substantial burden, particularly immediate family members closely involved in care. Burden is associated with both caregiving intensity and family support. Targeted interventions to support close family caregivers are warranted. Given the cross-sectional design, findings indicate associations rather than causality; longitudinal studies are needed to explore the psychological impact of sustained caregiving.

## 1 Introduction

According to the World Health Organization (WHO), the trend of an aging world population has brought with it an increased prevalence of chronic diseases among older adults, which has in turn led to an increased reliance on informal (typically family member) caregiving (1). The family caregiver burden is a multidimensional concept that includes the physical, emotional, social, and economic difficulties associated with caring long-term for older adults living with chronic health conditions (2). The adverse consequences of the accumulated strain caused by the responsibilities of maintaining care have been associated with a range of problems for family caregivers, including depression, anxiety and poorer quality of life (3), as well as higher rates of institutionalization for older adults.

Although caregiver burden is currently recognized as an emerging public health issue globally, research on this subject is necessary in the Arab world. Previous literature has emphasized the importance of further work on the caregiver burden and the factors, including sociocultural and health-related ones, that contribute to its development (4, 5). The determinants of caregiver burden are well known in geriatric nursing. They are related to the severity of the illness of the older adult, cognitive and behavioral functioning impairments, and the health condition and availability of family support for the caregiver (2, 3, 6).

The Zarit Burden Interview (ZBI) is the most frequently used instrument for measuring caregiving burden and it has been translated into several languages, including Arabic (7, 8). Studies in Saudi Arabia and the Gulf region have reported a continued association between high caregiver burden and female caregivers (9), those delivering substantial hours of care, and those with poor social or family history support. Evidence-based interventions such as formal caregiver training, respite care, and peer support groups have been found to be effective in reducing caregiver burden but are not widely available across many low- and middle-resource settings (10, 11).

With increasing demand for informal caregiving practice in Saudi Arabia and the paucity of local studies exploring caregiver burden, there is a strong rationale for studying the degree and covariates of the burden experienced by family caregivers of older adults with chronic diseases. Recognition of these determinants is important for guiding future interventions, policy development and resource allocation in a way that can improve caregiver well-being and sustain home care. Thus, the purpose of this study is to evaluate the burden of the caregivers of elderly relatives with chronic diseases and identify the demographic, clinical and psychosocial factors related to that.

## 2 Methods

### 2.1 Study design

A descriptive cross-sectional study was conducted among family caregivers who provide care for older adults with chronic diseases in Saudi Arabia.

### 2.2 Study population and sampling

The study focused on family caregivers of older adults (aged ≥60 years) with at least one chronic condition diagnosed, such as diabetes mellitus, cardiovascular disease, hypertension and dementia or Alzheimer Disease. The participants were recruited from outpatient units of King Fahad Hospital and Prince Meshari Hospital in Al-Baha region.

Convenience sampling among those who were presented to hospital was employed. Eligible caregivers who were attending the older adult to an outpatient visit at the time of data collection and during recruitment efforts encountered them and were invited to participate.

### 2.3 Inclusion criteria

Participants who are responsible for providing the majority of care to an older adult (≥60 years) diagnosed with at least one chronic disease, aged ≥18 years and have provided care for at least six months.

### 2.4 Exclusion criteria

The study excluded paid caregivers.

### 2.5 Sample size

The sample size was estimated using Steven K. Thompson’s formula (12), considering a 95% confidence level (Z = 1.96) and a 5% margin of error (d = 0.05). The parameter N represents the estimated population size of eligible family caregivers attending the outpatient clinics during the study period, based on hospital records. Based on these assumptions, the minimum required sample size was calculated as 140 participants. The final sample included 132 caregivers, yielding a response rate of 94.3%.


$$n=\frac{\left[N\times p(1-p)\right]}{\left[[N-1\times (\frac{d^{2}}{z^{2}})]+p(1-p)\right]}$$

The slight shortfall from the target sample size was due to non-response and refusal to participate during data collection.

### 2.6 Data collection tool

The study utilized an adapted questionnaire consisting of the following sections:

- Demographic characteristics of caregivers and older adults: Age, gender, education, employment, relationships to the patient, duration and hours of caregiving, type and number of chronic diseases, support systems.
- Zarit Burden Interview (ZBI): Caregiver burden was assessed using the validated Arabic version of the Zarit Burden Interview – Short Form (ZBI-12). The instrument consists of 12 items scored on a 5-point Likert scale ranging from 0 (never) to 4 (nearly always), with a total possible score ranging from 0 to 48, where higher scores indicate greater caregiver burden. Based on previously reported categorizations in the literature, scores were classified as 0–10 (no to mild burden), 11–20 (mild to moderate burden), and >20 (high burden) (7, 8).
- Social Support Scale: Perceived social support was measured using the Multidimensional Scale of Perceived Social Support (MSPSS). The MSPSS consists of 12 items assessing support from family, friends, and significant others, each rated on a Likert scale. The total score reflects the level of perceived social support, with higher scores indicating adequate support. For this study, total scores were categorized as follows: 12–48 = inadequate support and 49–84 = adequate support (13).

### 2.7 Validity and reliability of tool

Caregiver burden was assessed by the Zarit Burden Interview (ZBI). The ZBI is a well-established, validated scale for assessing perceived burden among family caregivers of older adults with strong construct validity and internal reliability (Cronbach’s alpha = 0.87–0.91 in prior research). The present study also reported the content validity and pilot of the tool, whose contents were reviewed and revised by five nursing experts and had satisfactory reliability (Cronbach’s α = 0.84). Another set of sociodemographic and caregiving information was obtained using a standard questionnaire developed from literature review and reviewed by experts for sequence, clarity and understanding. These processes have contributed to making the instruments valid and reliable, consequently increasing the credibility of findings in this study.

### 2.8 Data collection procedure

The researcher collected data from caregivers attending the King Fahad Hospital and Prince Meshari Hospital in Al-Baha. Written consent was obtained from the caregivers before they filled in the questionnaire.

### 2.9 Statistical analysis

The demographics and caregiving characteristics of the sample, such as age, gender, education level, occupation status, marital state; relationship to the care recipient; and type and number of chronic illnesses for older adults were described using descriptive statistics. Caregiver burden and family support were graphically displayed in graphs with distributions for categories of burden and levels of family support depicted based on separate figures. Inferential statistics were used to analyze the relationship between caregiver burden and related factors. The association of caregiver burden with family support and sociodemographic characteristics of caregivers was tested using Chi-square tests. For contingency tables, Chi-square tests were used when all expected cell counts were ≥5, and Fisher’s exact test was applied when expected counts were <5 to meet test assumptions. The ordinal logistic regression analysis was performed with predictors being age and sex, duration of caregiving, relationship to the care-recipients and number of chronic conditions in older people. The proportional odds assumption for the ordinal logistic regression model was assessed using the Test of Parallel Lines in SPSS. A p value < 0.05 was considered as statistically significant for all evaluations.

### 2.10 Ethical considerations

This study was ethically approved by the Ethical Committee of Deanship of Innovation and Scientific Research at Al-Baha University on 26/08/2025 with approval number 47103349. Written consent was signed by all the participating caregivers who had been informed about the study, including its aims, procedures, risks and the fact that participation was entirely voluntary. The research protocol complies with the principles of the Declaration of Helsinki, respecting participants’ autonomy, confidentiality and well-being at every stage.

## 3 Results

### 3.1 Summary of findings

**Table 1** shows the demographic characteristics of 132 family caregivers of elderly relatives with chronic diseases in Al-Baha, Saudi Arabia. The greatest proportion of caregivers were 20–39 years old (66, 50.0%), followed by those 40–59 years old (30, 22.7%). A smaller number were younger than 20 years old (12, 9.1%). The majority of the caregivers were women (114, 86.4%), and most lived in households of five (48, 36.4%) or six members (36, 27.3%). Over half had a university education (72, 54.5%) and half were unemployed (66, 50.0%). Most of the caregivers were unmarried (84, 63.6%) and the majority were either sons/daughters (54, 40.9%) or grandchildren (54, 40.9%) of the person receiving care.

**Table 1:** Demographic characteristics of family caregivers of older adults with chronic diseases in Al-Baha, Saudi Arabia (n=132).

Variables	Frequency	Percentage
Age		
18-< 20 years	12	9.1
20–39 years	66	50.0
40–59 years	30	22.7
60 years or more	24	18.2
Gender		
Male	18	13.6
Female	114	86.4
Family members		
Two	6	4.5
Three	6	4.5
Four	6	4.5
Five	48	36.4
Six	36	27.3
Seven	18	13.6
Ten	12	9.1
Educational levels		
Illiterate	6	4.5
Primary	12	9.1
Intermediate	18	13.6
Secondary	12	9.1
University	72	54.5
Postgraduate	12	9.1
Occupation		
Employed	24	18.2
Unemployed	66	50.0
Retired	18	13.6
Other	24	18.2
Marital status		
Single	84	63.6
Married	30	22.7
Divorced	6	4.5
Widowed	12	9.1
Relation with patient		
Son/daughter	54	40.9
Wife/husband	12	9.1
grandchildren	54	40.9
Other	12	9.1

**Table 2** presents the demographic characteristics of the care recipients (older adults). The most common age group was 70–79 years (84, 63.6%) followed by those aged ≥80 years (42, 31.8%). A total of 90 (68.2%) care recipients were female, while 42 (31.8%) were male. The majority of older adults were partially dependent on caregivers (78, 59.1%) and 42 (31.8%) presented behavioral or psychiatric symptoms. Regarding the duration of daily care, 48 (36.4%) older adults received 1 hour of care per day, whereas 24 (18.2%) received approximately 3 hours of care daily. The most common chronic conditions reported were hypertension (31.8%), Alzheimer’s disease (22.7%), and coexisting diabetes and hypertension.

**Table 2:** Demographic characteristics of older adults with chronic diseases received care from family caregivers in Al-Baha, Saudi Arabia (n=132).

Variables	Frequency	Percentage
Age of older adult		
60–69 years	6	4.5
70–79 years	84	63.6
80 years or more	42	31.8
Gender of older adult		
Male	42	31.8
Female	90	68.2
Degree of dependency on caregiver		
Dependent	18	13.6
Independent	36	27.3
Partial help	78	59.1
Behavioral and psychological problems of older adult		
Yes	42	31.8
No	90	68.2
Duration of daily care		
1 hour	48	36.4
2 hours	60	45.5
3 hours	24	18.2
Chronic diseases of older adult		
Hypertension	42	31.8
Alzheimer/Dementia	30	22.7
Diabetes Mellitus, Hypertension	18	13.6
Diabetes Mellitus, Hypertension, Alzheimer	12	9.1
Diabetes Mellitus, Hypertension, Renal diseases	12	9.1
Diabetes Mellitus	6	4.5
Diabetes Mellitus, Hypertension, Heart Diseases	6	4.5
Hypertension, Heart Diseases	6	4.5

**Figure 1:**
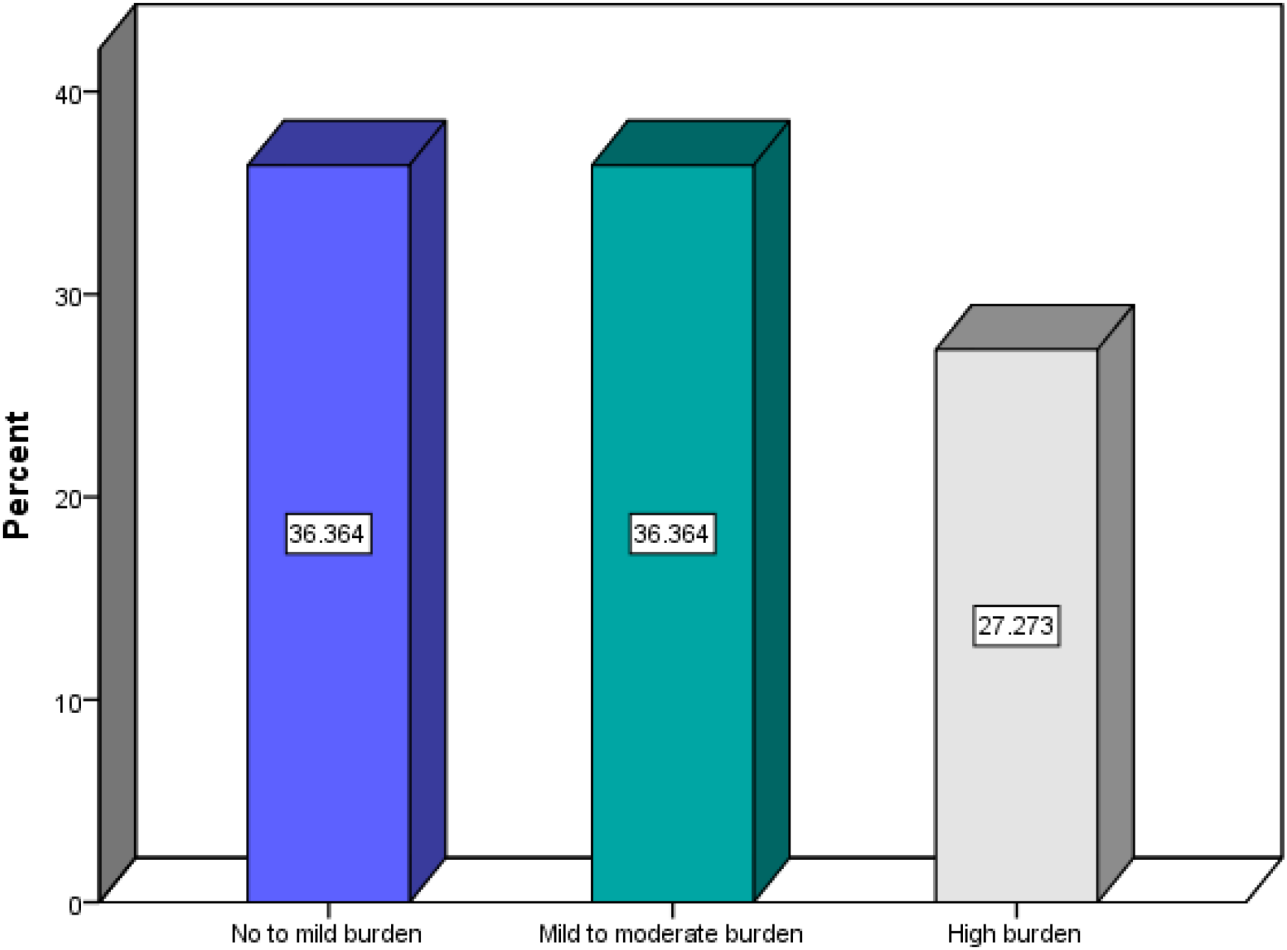
Level of caregivers’ burden in caring for older adults with chronic disease in Al-Baha, Saudi Arabia (n=132).

**Figure 1** presents the distribution of caregiver burden levels among family caregivers of older adults with chronic diseases in Al-Baha, Saudi Arabia (n = 132). The categories represent “No-to-mild burden” (score 0–10), “Mild-to-moderate burden” (score 11–20), and “High burden” (score >20) based on the 12-item Arabic Zarit Burden Interview. Percentages indicate the proportion of caregivers in each burden category.

**Figure 2:**
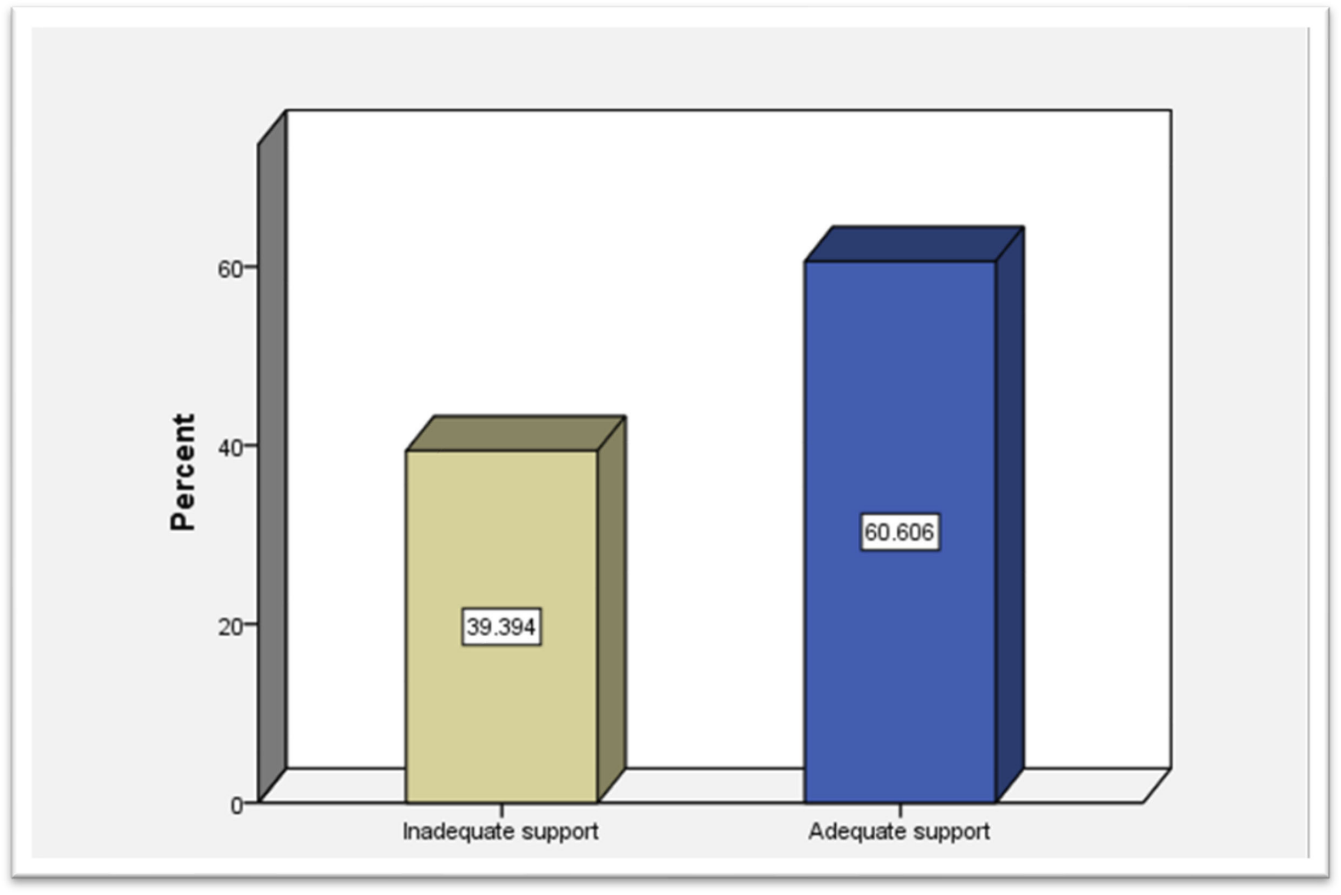
Level of support for caregivers in caring for older adults with chronic disease in Al-Baha, Saudi Arabia (n=132).

**Figure 2** presents the distribution of family support levels among caregivers of older adults with chronic diseases in Al-Baha, Saudi Arabia (n = 132). Support levels are categorized as “Low support” (score 0–10) and “High support” (score >10) based on the adapted social support scale. Percentages indicate the proportion of caregivers experiencing each level of support.

**Table 3** shows a significant relationship between family support and caregiver burden (p =. 001). Of caregivers with inadequate support, 27 (51.9%) reported a high burden, while 9 (11.2%) of those with adequate support reported a high burden. On the other hand, caregivers with sufficient support were less likely to perceive moderate to high burden (40, 50.0%) compared to those who have inadequate support.

**Table 3:** Association between family support for caregiver and the level of burden in caring for older adults with chronic disease in Al-Baha, Saudi Arabia (n=132).

Variables		Level of caregiver burden						p
		No to mild burden		Mild to moderate burden		High burden		
		Freq.	%	Freq.	%	Freq.	%	
Level of family support	Inadequate support	8	15.4%	17	32.7%	27	51.9%	<.001*
	Adequate support	40	50.0%	31	38.8%	9	11.2%	

(*) significant.

**Table 4** shows the relationship between demographic variables and caregiver burden. In these analyses, gender, age, education, and relationship with the care recipient showed significant associations in unadjusted comparisons. For example, female caregivers appeared more likely to experience higher burden than male caregivers (p < 0.001, Fisher’s exact test), and immediate family members (sons/daughters) reported higher burden compared to more distant relatives (p < 0.001, Fisher’s exact test).

**Table 4:** Association between family support for caregiver and the level of burden in caring for older adults with chronic disease in Al-Baha, Saudi Arabia (n=132).

Variables	Level of caregiver burden						p	Test used
	No to mild		Mild to moderate		High			
	Freq.	%	Freq.	%	Freq.	%		
Age								
< 20 years	0	0.0%	6	50.0%	6	50.0%	<.001*	Chi-square
20–39 years	36	54.5%	24	36.4%	6	9.1%		
40–59 years	6	20.0%	6	20.0%	18	60.0%		
> 60 years	6	25.0%	12	50.0%	6	25.0%		
Gender								
Male	0	0.0%	12	66.7%	6	33.3%	<.001*	Fisher’s exact
Female	48	42.1%	36	31.6%	30	26.3%		
Educational levels								
Illiterate	6	100.0%	0	0.0%	0	0.0%	<.001*	Fisher’s exact
Primary	0	0.0%	6	50.0%	6	50.0%		
Intermediate	0	0.0%	6	33.3%	12	66.7%		
Secondary	0	0.0%	6	50.0%	6	50.0%		
University	42	58.3%	24	33.3%	6	8.3%		
Postgraduate	0	0.0%	6	50.0%	6	50.0%		
Occupation								
Employed	6	25.0%	12	50.0%	6	25.0%	<.001*	Fisher’s exact
Unemployed	24	36.4%	24	36.4%	18	27.3%		
Retired	6	33.3%	12	66.7%	0	0.0%		
Other	12	50.0%	0	0.0%	12	50.0%		
Marital status								
Single	36	42.9%	36	42.9%	12	14.3%	<.001*	Fisher’s exact
Married	6	20.0%	12	40.0%	12	40.0%		
Divorced	0	0.0%	0	0.0%	6	100.0%		
Widowed	6	50.0%	0	0.0%	6	50.0%		
Relation with patient								
Son/daughter	6	11.1%	30	55.6%	18	33.3%	<.001*	Fisher’s exact
Wife/husband	0	0.0%	0	0.0%	12	100.0%		
grandchildren	30	55.6%	18	33.3%	6	11.1%		
Other	12	100.0%	0	0.0%	0	0.0%		

(*) significant.

**Table 5** shows the ordinal logistic regression analysis examining factors associated with caregiver burden among family caregivers in Al-Baha, Saudi Arabia (n = 132). After adjusting for potential confounders—including caregiver age, gender, occupation, marital status, education, and relationship with the care recipient—only the caregiver’s relationship with the patient remained a statistically significant predictor of burden (OR = 22.48, 95% CI = 4.09–123.46, p < 0.001). Gender and other sociodemographic factors were no longer significant, indicating that the initial bivariate associations were likely confounded by stronger predictors, particularly relationship closeness. These results suggest that proximity of familial relationships, rather than general demographic characteristics, is the primary determinant of caregiver burden in this population.

**Table 5:** Ordinal logistic regression analysis of factors associated with caregiver burden in Al-Baha, Saudi Arabia (n=132).

Variable	B	SE	Wald	p-value	OR (Exp B)	95% CI
Relationship (Category 1 vs reference)	3.113	0.870	12.814	<0.001	22.48	4.09–123.46
Occupation (Unemployed)	-1.072	0.665	2.603	0.107	0.34	0.09–1.26
Age	NS	—	—	>0.05	—	—
Gender	NS	—	—	>0.05	—	—
Marital Status	NS	—	—	>0.05	—	—
Education	NS	—	—	>0.05	—	—

OR, Odds Ratio; CI, Confidence Interval.

## 4 Discussion

The findings of this study have significant implications regarding the care burden experienced by family caregivers who care for elderly patients with chronic diseases. They demonstrate that caregiver strain is still a strong and continuous concern in geriatric healthcare. The current study confirms that caregiver burden is affected by many factors, such as the amount of care provided, the extent of impairment (functional and cognitive) in the care recipient and the psychosocial resources of caregivers. These results are in line with global evidence which shows that providing care for people living with chronic disease and age-related conditions puts a lot of physical, psychological and social stress on family members (14–18). The present findings provide further evidence that caregivers who deliver more hours of care and those caring for highly dependent older people are more likely to experience high levels of burden.

Relative to regional studies from Saudi Arabia and other countries in the Gulf, our results are consistent with those indicating that female caregivers, low family support, and patients with more complex care needs are at higher risk of experiencing a burden. For instance, reports carried out by Alshammari et al. (2017; 2023) also found that caregiver burden is higher when family support is low and care demands are high, especially when women are the main caregivers (4, 9). Nevertheless, the current study adds a level of complexity to this model by showing that demographic, clinical, and psychosocial factors work as predictors of caregiver burden, further solidifying the assertion that caregivers experience load not just from their role-related tasks but also through aggravating factors such as emotional self-efficacy and context-based support (19, 20). This is in line with global results reported by Adelman et al. and Kim et al., who found caregiver mental health and perceived support as key correlates of burden (2, 6). However, this is inconsistent with some studies in the Western world where access to formal care services was observed to reduce the burden of caregivers (21, 22).

Furthermore, the current study’s results suggest the utility of the ZBI for assessing burden levels in Arabic-speaking populations. Although the ZBI has been well tested internationally, moreover the results from Al-Rawashdeh et al. study emphasizes its cultural relevance in the Arab world (7). The consistency of finding in different settings emphasizes the universality of caregiver burden and also underscores contextual variations; most Arab countries do not have formalized caregiver support schemes, such as respite care, training programs, and caregiver support services. If established in the Arab region, these may lessen the burden of caregivers. Taken together, these comparisons emphasize the necessity of interventions and policies at a regional level that acknowledge the vital contribution of informal caregivers and offer them more structured and formal support.

This study highlights that immediate family members providing care to older adults with chronic diseases in Al-Baha, Saudi Arabia, experience the highest burden. While bivariate analyses indicated associations between gender, age, and education with burden, multivariable modeling clarified that these effects were confounded by relationship with the care recipient. The findings support the stress-process model of caregiving proposing that caregiver burden is a result of primary stressors (e.g., dependency level, care recipient behavioral symptoms), secondary stressors (e.g., role strains and conflicts) and coping resources available (e.g. family support). Thus, in this context, the closer to care recipient a caregiver is, the more intense the primary stressor is. This explains why sons and daughters were at highest risk of burden.

There are several limitations to this study. First, the cross-sectional design does not allow inference on causal relationships of caregiver characteristics with burden. Second, convenience sampling from two outpatient clinics in the Al-Baha region may introduce sampling bias and limit generalizability of the findings. A third limitation is that there were low numbers of participants in certain subgroups, which could have resulted in unstable estimates for the ordinal logistic regression. Lastly, data were self-reported and a potential recall bias or social desirability is suspected. Nevertheless, the study has its significance to gain an understanding of risk factors for caregiver burden and to consider a tailored caring support for family caregivers in older adults with chronic diseases.

## 5 Conclusions

Caregiver burden was prominent among family caregivers of older adults with chronic diseases in Al-Baha, Saudi Arabia. Burden was associated with factors related to demographic characteristics, clinical aspects, and psychosocial factors. Long-term care of older relatives with chronic illnesses may usefully screen for and address anxiety, depression, and other mental health problems in family caregivers. The results indicate that more hours spent providing care, higher functional disability of the elderly person being cared for and lower family support levels are associated with a higher burden among caregivers. This indicates the importance of systematic evaluation and early detection of caregivers at risk. Enhancing caregiver support interventions is crucial for promoting caregivers’ mental health outcomes and the well-being of both older adults and their caregivers.

## 6 Recommendations

It is suggested that as routine practice, healthcare systems should consider screening caregiver burden in geriatrics and chronic disease services, implement formal programs for structured education and other forms of psychosocial support for caregivers, increase access to preventive care options, and increase the availability of community-based/online support groups. It is recommended that policymakers place greater emphasis on developing formal caregiver support protocols that target the multidimensional caregiver burden. Researchers are also encouraged to design larger, longitudinal studies that examine the impact on caregiver burden of interventions that promote evidence-based practices in the Arab region and to conduct future studies using probability sampling methods and multi-center recruitment across different regions to improve representativeness and external validity. It is also recommended that future studies examine caregiver burden stratified by disease type (e.g., dementia, cardiovascular disease, mobility impairment) and level of dependency in activities of daily living and to investigate cross-cultural differences in caregiver burden and coping strategies to be conducted in the context of Saudi Arabia.

## Data Availability

The raw data supporting the conclusions of this article will be made available by the authors, without undue reservation.

## References

[1] World Health Organization . Ageing and health. (2024) Available online at: https://www.who.int/news-room/fact-sheets/detail/ageing-and-health (Accessed May 12,2025).

[2] Adelman RD Tmanova LL Delgado D Dion S Lachs MS . Caregiver burden: a clinical review. Jama. (2014) 311:1052–60. doi: 10.1001/jama.2014.304, PMID: 24618967

[3] Lindeza P Rodrigues M Costa J Guerreiro M Rosa MM . Impact of dementia on informal care: a systematic review of family caregivers’ perceptions. BMJ Supp Palliat Care. (2020) 14:e38–49. doi: 10.1136/bmjspcare-2020-002242. PMID: 33055092

[4] Alshammari B Noble H McAneney H Alshammari F O’Halloran P . (2023). “ Caregiver burden in informal caregivers of patients in Saudi Arabia receiving hemodialysis: a mixed-methods study”, in: Healthcare (Switzerland: MDPI). p. 366. 10.3390/healthcare11030366 PMC9914672 36766941

[5] Hüseyinsinoğlu BE Zirek E Aytutuldu GK Küçükoğlu H . Caregiver burden in caregivers of acute stroke patients: from a biopsychosocial perspective in a Turkey sample. Clin Exp Health Sci. (2021) 11:667–73. doi: 10.33808/clinexphealthsci.685431

[6] Kim H Chang M Rose K Kim S . Predictors of caregiver burden in caregivers of individuals with dementia. J Adv Nurs. (2012) 68:846–55. doi: 10.1111/j.1365-2648.2011.05787.x. PMID: 21793872

[7] Al-Rawashdeh SY Lennie TA Chung ML . Psychometrics of the Zarit Burden Interview in caregivers of patients with heart failure. J Cardiovasc Nurs. (2016) 31:E21–8. doi: 10.1097/jcn.0000000000000348. PMID: 27617563 PMC5069100

[8] Zarit SH Reever KE Bach-Peterson J . Relatives of the impaired elderly: correlates of feelings of burden. Gerontologist. (1980) 20:649–55. doi: 10.1093/geront/20.6.649. PMID: 7203086

[9] Alshammari SA Alzahrani AA Alabduljabbar KA Aldaghri AA Alhusainy YA Khan MA . The burden perceived by informal caregivers of the elderly in Saudi Arabia. J Family Community Med. (2017) 24:145–50. doi: 10.4103/jfcm.jfcm_117_16. PMID: 28932158 PMC5596626

[10] Chen H Makhdzir N Loh WC Wang L . Quality of life of family caregivers of stroke patients: a systematic review of qualitative research. Pakistan J Med Sci. (2025) 41:1211. doi: 10.12669/pjms.41.4.11136. PMID: 40290234 PMC12022594

[11] Chiao CY Wu HS Hsiao CY . Caregiver burden for informal caregivers of patients with dementia: a systematic review. Int Nurs Rev. (2015) 62:340–50. doi: 10.1111/inr.12194. PMID: 26058542

[12] Thompson SK . Sampling. New York City, USA: John Wiley & Sons (2012) 283–310.

[13] Zimet GD Dahlem NW Zimet SG Farley GK . The multidimensional scale of perceived social support. J Pers Assess. (1988) 52:30–41. doi: 10.1037/t02380-000. PMID: 2280326

[14] Lim J Zebrack B . Caring for family members with chronic physical illness: a critical review of caregiver literature. Health Qual Life Outcomes. (2004) 2:50. doi: 10.1186/1477-7525-2-50. PMID: 15377384 PMC521496

[15] Bífárìn O Quinn C Breen L Wu C Ke M Yu L . Stressors and coping mechanisms of family care-givers of older relatives living with long-term conditions in mainland China: a scoping review of the evidence. Ageing Soc. (2023) 43:952–89. doi: 10.1017/S0144686X21000817, PMID: 41292463

[16] Cormican O Meskell P Dowling M . Psychosocial vulnerability among carers of persons living with a chronic illness: a scoping review. Int J Nurs Pract. (2022) 28:e13024. doi: 10.1111/ijn.13024. PMID: 34741488

[17] Jika BM Khan HT Lawal M . Exploring experiences of family caregivers for older adults with chronic illness: a scoping review. Geriat Nurs. (2021) 42:1525–32. doi: 10.1016/j.gerinurse.2021.10.010. PMID: 34735999

[18] Phillips R Durkin M Engward H Cable G Iancu M . The impact of caring for family members with mental illnesses on the caregiver: a scoping review. Health Promo Int. (2023) 38:daac049. doi: 10.1093/heapro/daac049. PMID: 35472137 PMC10269136

[19] Sajadi SA Ebadi A Moradian ST Akbari R . Designing and validation of health-related quality of life inventory for family caregivers of hemodialysis patients. Int J Community Base Nurs Midwifery. (2020) 8:164. doi: 10.30476/IJCBNM.2020.83081.1118, PMID: 32309457 PMC7153424

[20] Wu J Li C Wu X Su D . Self‐efficacy and e‐health literacy among caregivers of patients with lung cancer: the chain‐mediating roles of negative emotions and caregiver readiness. J Clin Nurs. (2025). doi: 10.1111/jocn.17807. PMID: 40369696

[21] Miyawaki A Kobayashi Y Noguchi H Watanabe T Takahashi H Tamiya N . Effect of reduced formal care availability on formal/informal care patterns and caregiver health: a quasi-experimental study using the Japanese long-term care insurance reform. BMC Geriat. (2020) 20:207. doi: 10.1186/s12877-020-01588-7. PMID: 32532253 PMC7291452

[22] Wagner AK Graves AJ Reiss SK LeCates R Zhang F Ross-Degnan D . Access to care and medicines, burden of health care expenditures, and risk protection: results from the World Health Survey. Health Policy. (2011) 100:151–8. doi: 10.1016/j.healthpol.2010.08.004. PMID: 20828854

